# Inflammatory Biomarkers in Diabetic Macular Edema

**DOI:** 10.3390/jcm15051949

**Published:** 2026-03-04

**Authors:** António Campos, Maria João Furtado, Ângela Carneiro, Angelina Meireles, Carlos Neves, António Francisco Ambrósio, Inês Leal, João Figueira, João Pedro Marques, José Henriques, Manuel Falcão, Nuno Gomes, Rita Flores, Rufino Silva, Bernardete Pessoa

**Affiliations:** 1 Department of Ophthalmology, Santo André Hospital, ULSRL, 2410-197 Leiria, Portugal; antonio.figueiredo@chleiria.min-saude.pt; 2 ciTechCare, Center for Innovative Care and Health Technology, Polytechnic Institute of Leiria, 2411-901 Leiria, Portugal; 3 Department of Ophthalmology, Unidade Local de Saúde de Santo António, 4099-001 Porto, Portugal; angelinameireles@gmail.com (A.M.); bbtpessoa@gmail.com (B.P.); 4 Institute for the Biomedical Sciences Abel Salazar, University of Porto, ICBAS, 4099-002 Porto, Portugal; 5 Department of Ophthalmology, Unidade Local de Saúde de São João, 4200-319 Porto, Portugal; amvgcarneiro@gmail.com (Â.C.); falcao.manuel@gmail.com (M.F.); 6 Cardiovascular R&D Center—UniC@RISE, Faculty of Medicine, University of Porto, 4169-007 Porto, Portugal; 7 Department of Ophthalmology, Unidade Local de Saúde de Santa Maria, 1649-028 Lisbon, Portugal; bombordo.seven@gmail.com (C.N.); inescardosoleal@gmail.com (I.L.); 8 University Ophthalmology Clinic, ISAMB, Faculty of Medicine, University of Lisbon, 1649-004 Lisbon, Portugal; 9 Faculty of Medicine, Coimbra Institute for Clinical and Biomedical Research (iCBR), University Coimbra, 3000-548 Coimbra, Portugal; afambrosio@fmed.uc.pt (A.F.A.); joaofigueira@oftalmologia.co.pt (J.F.); marquesjoaopedro@gmail.com (J.P.M.); rufino.silva@oftalmologia.co.pt (R.S.); 10 Center for Innovative Biomedicine and Biotechnology (CiBB), University Coimbra, 3004-531 Coimbra, Portugal; 11 Clinical Academic Center of Coimbra (CACC), 3000-548 Coimbra, Portugal; 12 Association for Innovation and Biomedical Research on Light and Image (AIBILI), 3000-548 Coimbra, Portugal; 13 Ophthalmology Department, Unidade Local de Saúde de Coimbra, 3004-561 Coimbra, Portugal; 14 IPR—Instituto Português de Retina, 1600-581 Lisbon, Portugal; jose.henriques@retinaplus.com; 15 Department of Surgery and Physiology, Faculty of Medicine, University of Porto, 4169-007 Porto, Portugal; 16 Braga Hospital, 4710-243 Braga, Portugal; nunolgomes@gmail.com; 17 Faculty of Medical Sciences, Nova Medical School, 1169-056 Lisbon, Portugal; ritamariaflores@gmail.com; 18 Ophthalmology Department, Unidade Local de Saúde de São José, 1150-199 Lisbon, Portugal; 19 Unit for Multidisciplinary Research in Biomedicine, Institute for the Biomedical Sciences Abel Salazar, University of Porto, (UMIB ICBAS—UP), 4050-313 Porto, Portugal

**Keywords:** diabetic retinopathy, diabetic macular oedema, OCT biomarkers, inflammation, inflammatory biomarkers

## Abstract

Diabetic retinopathy (DR) is a major complication of both Type 1 and Type 2 diabetes mellitus (T1DM and T2DM). Disease progression can result in visual impairment, primarily due to diabetic macular edema (DME) or proliferative diabetic retinopathy (PDR). Although several ocular treatments are available for DME, a subset of patients fails to respond, reflecting the multifactorial, complex, and systemic nature of DR. Inflammatory biomarkers can be classified according to different characteristics, including imaging biomarkers—most commonly assessed using optical coherence tomography (OCT)—and molecular biomarkers, which are defined by their biochemical and biophysical properties. Pro- and anti-inflammatory cytokines, chemokines, adipokines, and inflammation-related enzymes are recognized as key inflammatory biomarkers and can be detected in the vitreous humour, aqueous humour, tears, serum, and other biological tissues. The identification and characterization of reliable biomarkers may help determine disease severity, monitor disease progression, and predict the risk of specific outcomes, thereby aiding in the prevention of end-stage disease (prognostic biomarkers). In addition, biomarkers may serve as predictive tools for therapeutic response, guiding personalized treatment strategies and enabling ongoing monitoring. This review provides a comprehensive overview of the role of inflammatory biomarkers in the diagnosis and management of DR and DME.

## 1. Introduction

Diabetic retinopathy (DR) is a serious condition associated with both Type 1 and Type 2 diabetes mellitus (T1DM and T2DM). It remains the primary cause of vision loss and preventable blindness in the working-age population [1]. According to the International Diabetes Federation, approximately 537 million adults worldwide are currently living with diabetes, a figure projected to rise to about 643 million by 2030 [2]. Approximately one-third of individuals with diabetes develop DR, with an estimated 10% experiencing vision-threatening complications [3].

The retina constitutes a complex neurovascular unit in which glial, neuronal, and vascular cells interact to maintain tissue homeostasis [4,5]. Although DR has traditionally been regarded as a primarily vascular disorder, growing evidence highlights the critical contribution of neurodegeneration and chronic inflammation to its pathogenesis [6]. Despite the availability of several therapeutic options for diabetic macular edema (DME) and proliferative diabetic retinopathy (PDR)—including intravitreal anti-VEGF agents as the first-line treatment, with corticosteroids, laser photocoagulation, and vitreoretinal surgery as second-line strategies—substantial variability in treatment response persists, with real-world data showing suboptimal outcomes and patients being often undertreated [7,8].

Retinal imaging and circulating biomarkers may play a valuable role in identifying early disease states and risk of progression, therefore stratifying individuals to personalized therapeutic approaches [9]. Biomarkers derived from ocular fluids, such as the vitreous or aqueous humour, may also be informative in the context of DR; however, their collection typically requires invasive procedures, presenting significant practical challenges [6,10]. Advances in retinal imaging have significantly enhanced the diagnosis and management of DR and DME [11]. When incorporated into a multimodal imaging strategy, fluorescein angiography (FA) and high-resolution techniques—such as optical coherence tomography (OCT) and OCT angiography (OCTA)—may support the development of better monitoring and personalized treatment strategies for DR and DME [12,13].

Although research on biomarkers has been prolific, the results of the studies are far from consensual due to the lack of a strong body of evidence [14,15,16,17].

This review aims to examine the role of inflammatory biomarkers in the diagnosis and management of DR and DME, with a focus on their potential to enhance early detection and support personalized treatment strategies. It includes a critical view on the available evidence including on the value of biomarkers of DME other than OCT biomarkers and their value as predictive or prognostic.

## 2. Methods

We conducted a comprehensive narrative literature review focusing on inflammatory biomarkers relevant to the diagnosis and management of DR and DME. Studies were identified through a systematic search of the MEDLINE, Embase, and Web of Science databases, as well as the Association for Research in Vision and Ophthalmology (ARVO) Meeting Abstracts website. The following keywords were used: “Diabetic Macular Edema” AND “Inflammation”; “Diabetic Macular Edema” AND “Clinical Outcomes”; “Diabetic Retinopathy” AND “Inflammatory Biomarkers”; “Diabetic Macular Edema AND “Serum Biomarkers” AND “Aqueous Biomarkers” AND “Vitreous Biomarkers.” All relevant abstracts were screened, and eligible full-text articles were reviewed for inclusion by two of the authors (AC and BP). Searches were restricted to English-language articles in PubMed and ARVO, and to English, Spanish, Portuguese, and French articles in Web of Science and MedlinePlus^®^ (National Library of Medicine (US)). Available from: https://medlineplus.gov accessed [10 June 2024]. We also searched for guidelines and recommendations of ophthalmological societies and the proceedings of relevant meetings. The search was performed between June and October 2024, and updated subsequently on 17 December 2025. This narrative review included data from randomized controlled trials, cohort, observational and retrospective studies, case series and experimental studies.

### 2.1. The Role of Inflammation in Diabetic Retinopathy and Diabetic Macular Edema

**Figure 1:**
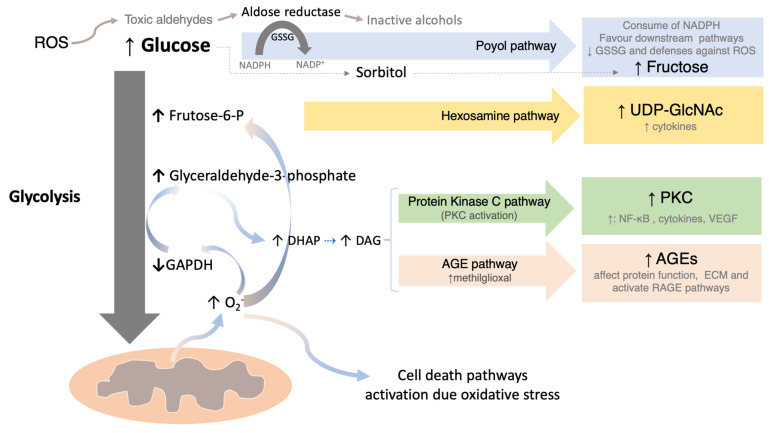
Hyperglycemia-induced mitochondrial superoxide overproduction activates four pathological pathways: the polyol, hexosamine, PKC, and AGE pathways. Data summarized from Nishikawa T et al., 2000 [23]; Brownlee M. 2001 [19]; Du XL et al., 2000 [15,24]. AGE = advanced glycation end products; DAG = diacyl glycerol; DHAP = dihydroxyacetone phosphate; ECM = extracellular matrix; GAPDH = glyceraldehyde-3-phosphate dehydrogenase; GSSG = glutathione; NADP^+^ = oxidized nicotinamide adenine dinucleotide phosphate; NADPH = reduced nicotinamide adenine dinucleotide phosphate; NF-κB = nuclear factor-kappa B; PKC = protein kinase C; RAGE = receptor for AGE; ROS = reactive oxygen species; UDP-GlcNAc = UDP-N-acetylglucosamine; VEGF = vascular endothelial growth factor. From Pessoa B, 2022 [25]. https://hdl.handle.net/10216/140033, accessed [20 June 2024]. Reproduced under permission.

Chronic hyperglycaemia is a major driver of retinal dysfunction in diabetes, triggering persistent low-grade inflammation mediated by mitochondrial superoxide overproduction resulting from excess intracellular glucose and metabolic overload [18]. This leads to an excess of electrons in the mitochondrial electron transport chain, inhibition of glyceraldehyde-3-phosphate dehydrogenase (GAPDH), and diversion of glycolytic intermediates into four pathogenic pathways: the polyol pathway, the hexosamine biosynthesis pathway, protein kinase C (PKC) activation, and the formation of advanced glycation end-products (AGEs). Reactive oxygen species (ROS) further exacerbate cellular injury by activating apoptotic pathways [19] (Figure 1). These deregulated pathways contribute to microvascular damage, neuronal apoptosis, and glial activation [20]. Cytokines are membrane-bound or soluble proteins that mediate cell-to-cell signalling and regulate a wide range of biological processes, including cell proliferation, inflammation, angiogenesis, immune responses, cell migration, fibrosis, tissue repair, ansd apoptosis. Pro-inflammatory cytokines include interleukin (IL)-1, IL-6, IL-17, tumour necrosis factor (TNF)-α, TNF-β, and placental growth factor (PlGF), whereas anti-inflammatory mediators include IL-1 receptor antagonist (IL-1Ra), IL-4, IL-10, IL-13, pigment epithelium-derived factor (PEDF), and angiopoietin-1 (Ang-1). Chemokines such as IL-8, monokine induced by interferon-γ (MIG/CXCL9), interferon-γ-induced protein 10 (IP-10/CXCL10), and RANTES (regulated upon activation, normal T cell expressed and secreted) promote leukocyte recruitment, amplify inflammatory signalling, and contribute to angiogenesis, fibrosis, and tissue homeostasis [21,22].

### 2.2. Biochemical Biomarkers of Inflammation in Diabetic Retinopathy and Diabetic Macular Edema

#### Vitreous and Aqueous Humour

Although various growth and inflammatory factors can be measured in both aqueous and vitreous humour, their concentrations do not always correlate [26]. The vitreous humour provides a more accurate reflection of retinal pathophysiology; however, its invasive collection limits routine use, except during vitrectomy procedures [27]. Several vitreous biomarkers are summarized in Table 1.

### 2.3. Angiogenic Factors

Key mediators in DR, including VEGF, IL-6, and IL-8, contribute to disease progression and are notably elevated in advanced stages such as PDR and DME [41,42,43,44]. Interestingly, vitreous VEGF levels do not correlate with serum VEGF, nor do plasma cytokine levels reflect those in the vitreous or aqueous humour [45,46,47]. However, VEGF concentrations in the aqueous and vitreous humours are correlated, including in patients with PDR [45,48]. Other relevant biomarkers in the vitreous of DR patients include PlGF, platelet-derived growth factor (PDGF), and PEDF [49,50,51]. PlGF supports neovascularization and endothelial growth [52], while PDGF is involved in central nervous system and retinal development, often upregulated in PDR [53]. PEDF has anti-angiogenic properties; low PEDF and high VEGF-A levels have been linked to DR progression, PDR development and refractory DME [53,54]. Additional biomarkers were identified in patients with PDR include angiopoietin-1 (Ang-1), angiopoietin-2 (Ang-2), matrix metalloproteinase-9 (MMP-9), and hepatocyte growth factor (HGF) [55]. Erythropoietin (EPO) appears to have a dual role, acting as pathogenic in advanced DR but protective during early stages [56,57,58].

### 2.4. Pro-Inflammatory Cytokines

Monocyte chemoattractant protein-1 (MCP-1/CCL2) recruits immune cells and may contribute to inflammation in vitreoretinal diseases. IL-6, IL-8, and MCP-1 are regulated by nuclear factor-kappa B (NF-κB), a key transcription factor in immune responses [59]. MCP-1 has been implicated in the progression of PDR and persistent post-vitrectomy DME, even in the absence of significant VEGF elevation [60].

IL-6 and IL-8 appear to be more strongly associated with the progression of DR to PDR [41].

### 2.5. Vitreous

Levels of adhesion molecules—including vascular cell adhesion molecule-1 (VCAM-1), intercellular adhesion molecule-1 (ICAM-1), E-selectin, and soluble vascular adhesion protein-1 (sVAP-1)—are elevated in the vitreous humour of PDR patients, contributing to leukostasis and vascular damage [61,62,63]. ICAM-1 correlates with leukocyte infiltration in the retina, while VCAM-1 and E-selectin may promote angiogenesis and correlate with vitreous VEGF levels [64].

Neurotrophins (NTs) and pro-inflammatory cytokines such as IL-1, IL-6, and IL-8 are also elevated in the vitreous humour of DR patients [65]. Vitreous concentrations of IL-6, IL-8, monokine induced by interferon-γ (MIG/CXCL9), and interferon-γ–induced protein 10 (IP-10/CXCL10) strongly correlate with DR severity and may serve as prognostic biomarkers following vitrectomy [41]. TNF-α, primarily produced by macrophages and T cells and regulated by NF-kB, is elevated in both serum and vitreous humour in DR patients [66,67]. Intraocular IL-6 is emerging as a potential therapeutic target due to its upstream role in regulating other cytokines and VEGF [68].

### 2.6. Aqueous

Diabetic patients exhibit elevated levels of IL-1β, IL-6, IL-8, MCP-1, IP-10, and VEGF, alongside reduced levels of IL-10 and IL-12 in the aqueous humour. Higher concentrations of cytokines in the aqueous humour—including IL-6, IL-8, IL-10, VEGF, transforming growth factor-beta (TGF-β), VCAM-1, ICAM-1, and MCP-1—are associated with disease severity, particularly in neovascular glaucoma and PDR [69].

Conversely, the concentrations of IL-1α, IL-4, IL-9, IL-21, IL-23, IL-27, IL-31, RANTES/CCL5, interferon-α, growth-regulated oncogene (GRO), and TNF-α are significantly lower in the aqueous humour of DR patients compared with control and non-proliferative DR groups [70].

### 2.7. Serum

Although several inflammatory molecules, such as TNF-α and IL-6, have been proposed as potential serum biomarkers in DR, these findings have not been consistently confirmed in larger studies [71]. C-reactive protein (CRP) seems to be increased in obese patients with DM with a body mass index > 30 kg/m^2^, which may be partly be due to adipocytes stimulating hepatic CRP production [72]. Notably, increased levels of high-sensitivity CRP (hsCRP) and ICAM-1 have been identified as potential biomarkers for the anatomical response to anti-VEGF therapy. Additionally, a neutrophil-to-lymphocyte ratio cut-off of 2.26 has been found to be a highly sensitive and specific predictor of DME [73].

Higher glycated hemoglobin (HbA1c) levels are associated with an increased risk of DR progression, and acute elevations may contribute to the development of DME. In fact, most patients with treatment-naïve DME have baseline HbA1c levels above 7%. Moreover, higher baseline HbA1c levels have been linked to a reduced therapeutic response [74,75,76].

### 2.8. Tear Fluid

Tear fluid may serve as an important source of biological material for evaluating ocular diseases using minimally invasive methods. Several studies have examined tear fluid biomarkers in patients with DME and DR (Table 2), revealing significant differences in tear protein profiles between patients with DR and healthy individuals [77,78].

### 2.9. Cellular Biomarkers

**Figure 2:**
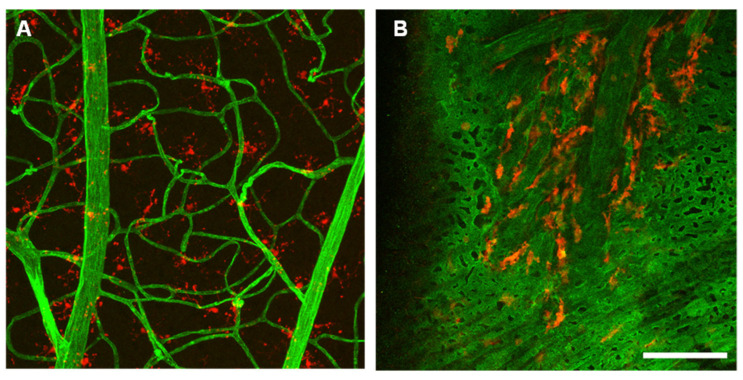
In vitro visualization of glial cells. (**A**) Presence of glial cells around vessels in the retina and in the choroid. Retinal projection showing vessels RECA1+ (green) and Iba+ cells/microglia (red) mostly around the retinal capillary plexus vicinity. (**B**) Choroid single confocal plane visualized from the RPE side, showing choroidal medium-sized and large vessels (green) surrounded by Iba+ cells (red). Laser scanning. From microscope LSM 710 (Zeiss, Oberkochen, Germany), objective lens: 20×, numerical aperture 0.8, magnification 200×. Scale bar = 50 μm. From Campos A, 2020 (doctoral thesis) and Campos et al., 2020 [82,93]. Accessed at https://www.researchgate.net/publication/346061663_Study_on_the_Contribution_of_the_Choroid_to_the_Pathophysiology_of_Diabetic_Retinopathy [accessed 20 September 2024]. Reproduced under permission. Licence CC BY-NC-SA 4.

**Figure 3:**
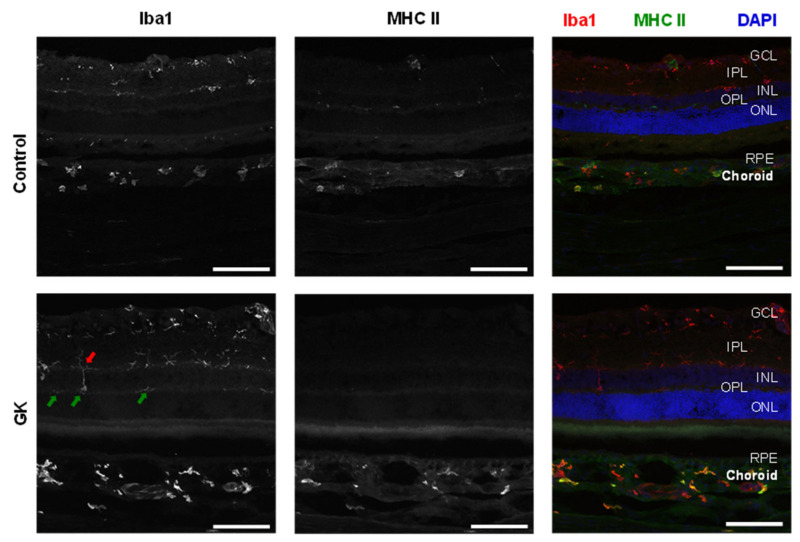
Microglial cells in the retina and choroid of GK (**bottom**) and age-matched control 52 W Wistar Han rats (**top**). Representative eye cross-sections immunolabeled against Iba1 (**left panels**), MHC II (**middle panels**), and merged (**right panels**). Iba1+ cells are located in the superficial and plexiform layers of the retina in controls. Iba1+ cells located in the OPL of the GK cohort only (green arrows). In GK rats, Iba1+ cells migrate from the IPL to the OPL, crossing the INL (red arrow). GCL = ganglion cell layer; IPL = inner plexiform layer; INL = inner nuclear layer; OPL = outer plexiform layer; ONL = outer nuclear layer; RPE = retinal pigment epithelium. Scale bar: 100 μm. From Campos et al., 2020 [82]. Reproduced under permission CC BY-NC-ND 4.0.

Cellular biomarkers cannot be evaluated in vivo in humans; however, data from diabetic animal models provide valuable insights into the pathophysiology of DR. These findings can be extrapolated to clinical data obtained through OCT [81,82,83]. Despite the fact that blood vessels are the only structures visible on clinical examination, they account for less than 5% of total retinal mass, while neurons, glial cells, and microglia account for over 95% [4]. Growing evidence suggests that Müller cells are functionally altered in DR and DME and may play a key role in disease progression [84]. Müller cells, as the primary glial cells of the retina, play a crucial role in retinal inflammation, integrity of the blood–retinal barrier (BRB), neuroprotection by segregating neurotrophic factors for the retinal neurons and regulation of macular water balance [85]. In fact, Müller cells are primarily involved in the response of retinal edema to steroids via the activation of potassium channels and aquaporin (AQP) 4 [86]. Müller cell depletion results in BRB disruption and vascular abnormalities, both key features of DR [87]. Vujosevic and colleagues reported significantly elevated levels of biomarkers of Müller cell activation, including glial fibrillary acidic protein, AQP1, and AQP4, in the aqueous humour of DR eyes [88]. Further evidence supporting the role of inflammation in the progression of DR is the association between vision-threatening stages of DR—including PDR, NPDR, and DME—and increased numbers of microglia, perivascular macrophages, and vitreal hyalocytes, in the retina and adjacent tissues [89]. As DR worsens, hyperglycemia activates microglial cells, which migrate across the retina and accumulate near the retinal capillary plexuses (Figure 2). These cells become detectable in vitro within the outer retinal layers and choroid, where they are typically rare or absent (Figure 3) [89]. The activation of microglia induces the release of various pro-inflammatory mediators, such as cytokines, chemokines, caspases, and glutamate, promoting cell proliferation and migration. This, in combination with decreased production of neurotrophic factors by the Müller cells, leads to an increase in neuronal apoptosis, thinning of the retinal nerve fibre layer, and dropout of the deep capillary plexus, ultimately leading to progressive vision loss [90].

Infiltration of glial cells into the retinal layers is frequently associated with the presence of hyperreflective foci (HRF) on OCT [82,91,92]. The accumulation of glial cells in the outer retina and choroid further exacerbates inflammation by releasing cytokines, impairing fluid clearance, and contributing to the development of DME [81].

### 2.10. Imaging Biomarkers of Inflammation in DR and DME

#### Fluorescein Angiography/Ultra-Widefield Fluorescein Angiography

**Figure 4:**
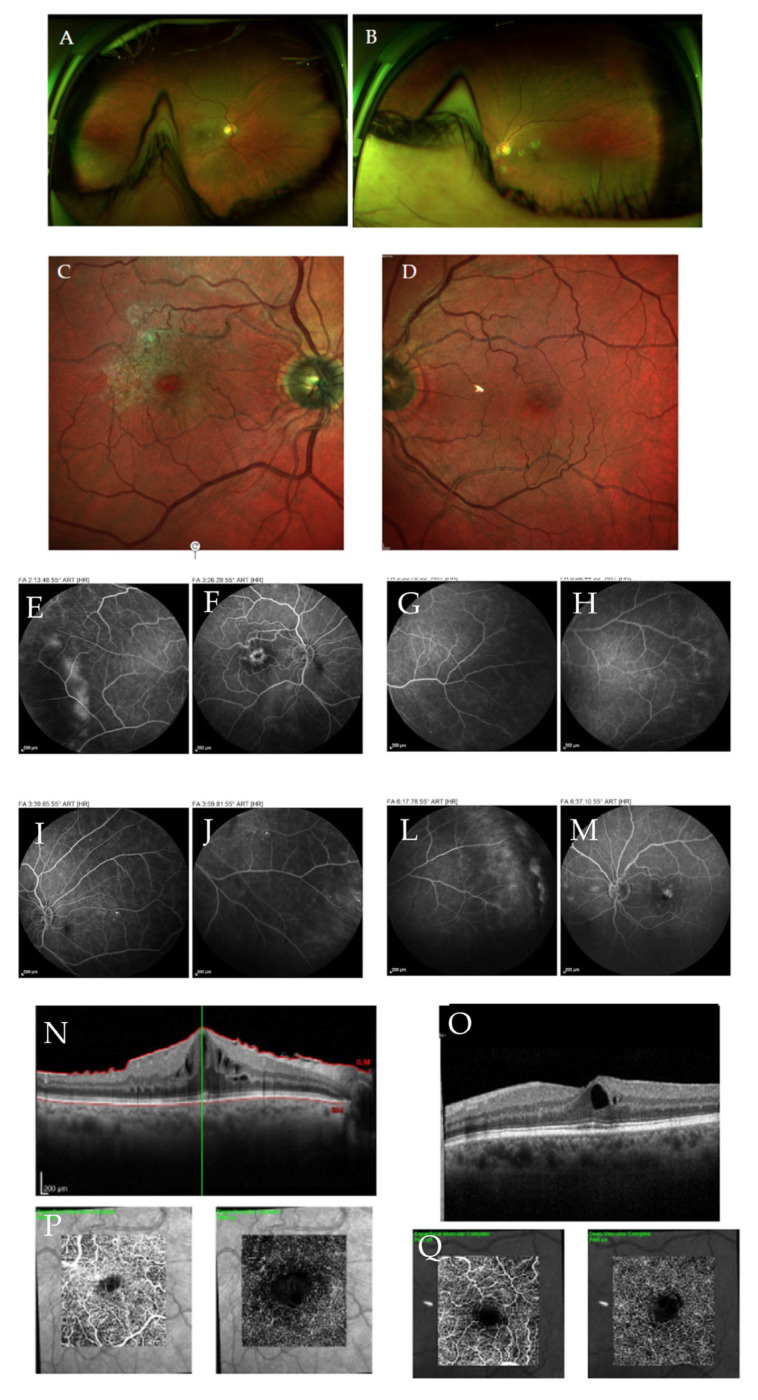
Multimodal approach to T2DM 62-year-old. No DR present in the CFP of both eyes (**A**,**B**). An ERM is present in the RE (**C**,**N**) but not in the LE (**D**,**O**), revealed on multicolor photo and OCT. FA images show peripheral PDR in the RE (**E**,**F**,**I**,**J**) and in the LE (**G**,**H**,**L**,**M**), and center-involvingmacular edema also visible in both eyes on OCT (**N**,**O**). In the 3 × 3 mm macular OCTA, the presence of microaneurysms in the deep capillary plexus and enlargement of the foveal vascular zone can also be observed in the RE (**P**), and although not as serious in the LE (**Q**). Although this patient had good metabolic control in the last two years, he had poor glycemic control in the four years prior to the diagnosis of DM. RE = right eye; LE = left eye; DR = diabetic retinopathy; ERM = epiretinal membrane; OCT = optical coherence tomography; OCTA = OCT-angiography; PDR = proliferative diabetic retinopathy; T2DM = Type 2 diabetes mellitus. Courtesy of Bernardete Pessoa, adapted from Henriques et al., 2023 [96].

FA remains the gold standard for evaluating the retinal vasculature [12]. It enables detection of neovascularization, retinal capillary non-perfusion, vascular telangiectasia, capillary dropouts, extension and irregularity of the foveal vascular zone (FAZ), and assessment of the peripheral retina [12]. Studies using ultra-widefield FA have shown that DR lesions are predominantly located outside the standard ETDRS (Early Treatment Diabetic Retinopathy Study) fields in 30–40% of the eyes. In addition, predominantly peripheral lesions (PPLs) have been associated with a higher level of severity in 9–15% of eyes and may increase the risk of progression of DR by 3 to 4 times [94,95]. PPLs are a biomarker of disease severity, particularly in patients with additional risk factors such as T1DM, prolonged diabetes duration, poor glycemic control, hypertension, obesity, dyslipidemia, nephropathy, insulin therapy, young age, short axial length, pregnancy, or history of ocular surgery (Figure 4) [96].

### 2.11. Fundus Autofluorescence

Fundus autofluorescence (FAF) is a rapid, non-invasive imaging technique, where lipofuscin in the retinal pigment epithelium (RPE) is its primary source of autofluorescence [97]. In DR, ocular inflammation and oxidative stress contribute to increased lipofuscin accumulation and reduced macular levels of lutein and zeaxanthin. Histological studies have shown that lipofuscin accumulates in microglial cells to a greater extent than in the RPE [98]. Vujosevic et al. hypothesized that the increased FAF areas observed in DME may result from the accumulation of oxidative by-products generated by activated microglia [99]. An alternative hypothesis attributes these changes to the mechanical effect of cystoid macular edema (CMO). Cysts are mostly located in the outer plexiform layer (OPL) and in the inner nuclear layer (INL), the layers with the highest concentration of lutein pigment. These cysts can displace macular pigment, thereby preventing the normal attenuation of the foveal FAF signal [99]. Although areas of increased FAF (iFAF) tend to decrease significantly after both steroid and anti-VEGF therapy, those with more extensive baseline iFAF appear to respond better to steroids [100].

### 2.12. Optical Coherence Tomography

**Figure 5:**
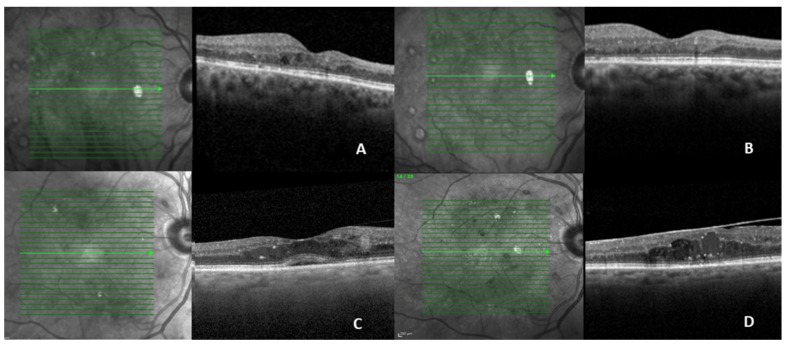
SD-OCT images representing, from baseline to one year of follow-up, two sub-types of patients: a good early responder to anti-VEGF (ranibizumab) therapy, with mild DME, with no INL cysts (**A** and **B** respectively) and a poor responder to anti-VEGF (ranibizumab) therapy (**C** and **D** respectively). In the latest example the HRF and sub-retinal fluid anticipate a more inflammatory and chronic sub-type of DME with progressive anatomic worsening and epiretinal membrane development over time, despite anti-VEGF monthly therapy (**D**). DME = diabetic macular edema; HRF = hyperreflective foci; INL = inner nuclear layer; SD-OCT = spectral-domain-optical coherence tomography; VEGF = vascular endothelial growth factor. Courtesy of Bernardete Pessoa. Adapted from Pessoa et al., 2024 [103].

**Figure 6:**
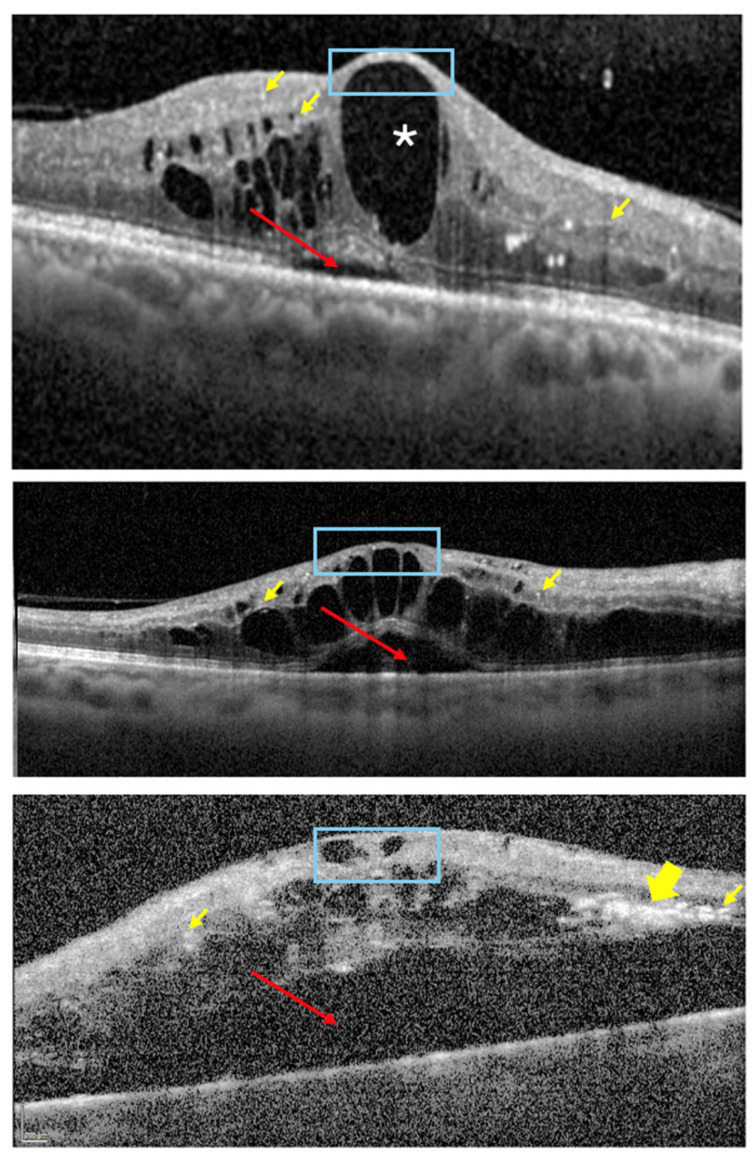
Three examples of SD-OCT biomarkers anticipating a more inflammatory and chronic DME with large cysts (top, white asterisk), DRIL (middle, blue square), HRF (thin yellow arrows), HE/confluent HRF (bottom, large yellow arrow) and SRF (red arrows). DME = diabetic macular edema; DRIL = disorganization of the retinal inner layers; HE = hard exudates; HRF = hyperreflective foci; SD-OCT = spectral-domain-optical coherence tomography; SRF = subretinal fluid. Courtesy of Bernardete Pessoa. Adapted from Henriques et al., 2023 [96].

OCT is an essential imaging modality for the diagnosis, management, and follow-up of patients with DR and DME (Table 3) [13,101]. The classification system for diabetic maculopathy proposed by the European School for Advanced Studies in Ophthalmology (ESASOs) stratifies the disease into early and advanced stages. The early stage is considered potentially reversible, while the advanced stage is associated with greater severity and limited reversibility. A third category—severe/atrophic maculopathy—represents an end-stage irreversible condition [102]. According to this classification, increased retinal thickness (>400 µm); disruption of the “E” layers (external limiting membrane and ellipsoid layer); increased number of HRF (>30); large (100–200 µm) intraretinal cysts, particular when associated with retinal bridging or located in the outer retina; the presence of subretinal fluid (SRF), especially if chronic; and a thickened vitreoretinal interface with complex epiretinal membranes, are all considered markers of a progressively severe and potentially cumulative inflammatory disease. Additional OCT biomarkers—such as INL cysts, OPL disruption, hard exudates, hyper-reflective cystoid walls, and a reduced choroidal vascular index—have also been associated with a more chronic pro-inflammatory state and worse anatomical and functional outcomes (Figure 5 and Figure 6) [101,103].

### 2.13. Hyperreflective Foci

HRF are detectable in diabetic eyes even before clinical detection of DR, and their prevalence increases with disease progression. They are thought to be associated with activation of microglia, infiltrating leukocytes, proteins, and/or lipid exudates, resulting from the breakdown of the inner BRB and reflecting an underlying inflammatory process. Eyes presenting these biomarkers seem to have a better anatomical and functional outcomes when treated with steroids [107].

### 2.14. Disorganization of the Inner Retinal Layers

The disorganization of the inner retinal layers (DRILs) was also referred as a predictive biomarker of functional outcomes. Specifically, the presence of DRIL was associated with a 7-fold increased risk of reduced best-corrected visual acuity (BCVA) following treatment. However, a universal accepted definition and quantification is lacking [108].

### 2.15. Disruption of the Ellipsoid Zone

Disruption of the ellipsoid zone (EZ) is associated with a 9- to 11-fold increased risk of reduced BCVA following therapy, reflecting outer retinal and photoreceptor damage [75,108]. Among all biomarkers, EZ disruption correlates most strongly with visual decline and is considered the most reliable predictor of advanced DR [108,109]. Despite its clinical relevance, no standardized method currently exists for grading EZ disruption. Some authors define severity based on horizontal or vertical scan extension (e.g., >100 µm within the central 1000 µm), while others apply thresholds of 200 µm or 500 µm, or consider any focal foveal involvement to be clinically significant [75,110,111].

### 2.16. Disrupted Cone Outer Segment Termination (COST Line) and Posterior Vitreous Detachment (PVD)

A disrupted COST line at baseline has been reported as a negative prognostic sign for future BCVA with a moderate certainty and a PVD has been associated with a better BCVA with a low certainty [112].

### 2.17. External Limiting Membrane

The external limiting membrane (ELM), formed by the junction between Müller cell processes and photoreceptor inner segments, is a key structural marker. Its disruption serves as a predictive biomarker of treatment response and correlates with photoreceptor damage and increased chronicity of intraretinal fluid (IRF), likely due to osmotic imbalance and fluid accumulation. It largely correlates and parallels EZ disruption [113,114,115,116,117,118].

### 2.18. Optical Coherence Tomography Angiography

Optical coherence tomography angiography enables early detection of advanced DR features, such as intraretinal microvascular abnormalities, capillary non-perfusion, and neovascularization, before they become clinically evident on colour fundus photography (CFP) or FA [119,120]. Key biomarkers of DR progression over a 5-year period include microaneurysm turnover and capillary dropout, particularly within the deep capillary plexus (DCP), which is the most affected vascular layer in DR [121,122]. DCP loss and OPL disruption have been associated with poor response to anti-VEGF treatment, poorer prognosis, often indicating better anatomic response to steroids [123].

**Figure 7:**
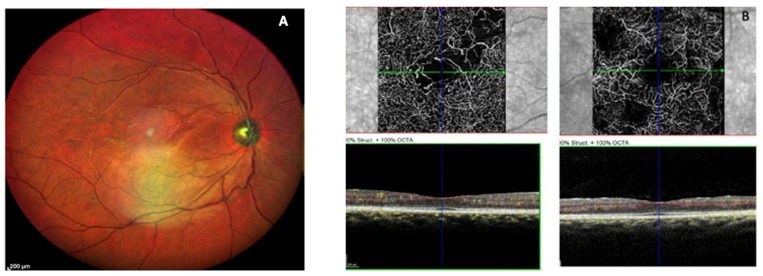
Right eye of a 26-year-old T1DM female, with mild NPDR, according to the ETDRS-DRSS classification ((**A**)—multicolor fundus photo). Despite clinical grading, OCTA reveals important macular ischemia, highlighting underlying microvascular compromise (**B**). NPDR = non proliferative diabetic retinopathy; OCTA = optical coherence tomography angiography; T1DM = Type 1 diabetes mellitus. Courtesy of Bernardete Pessoa. Adapted from Henriques et al., 2023 [96].

Other predictive OCTA parameters include FAZ morphology (size and circularity), microaneurysm characteristics (visibility, reflectivity, number, and location), vascular density, non-perfusion areas, and vessel tortuosity [124]. Within the DCP, hyperreflective microaneurysms, increased microaneurysm burden, reduced vascular density, and enlarged FAZ have all been associated with reduced response to anti-VEGF therapy (Figure 7) [123,125,126].

### 2.19. Correlation Between Inflammatory Biomarkers, DME and the Choice for Steroid Implants

Associations between aqueous humour cytokine levels and DME subtypes identified via OCT were reported [127]. VEGF and IL-6 were linked to sponge-like diffuse retinal thickening, while IL-6 and MCP-1 were associated with CMO.

A strong association between SRF and intravitreal IL-6 levels has been reported as well [128]. Patients with a greater inflammatory load exhibited better anatomical response to a dexamethasone implant, supporting the role of inflammation in DME. The presence of SRF, hyperreflective cysts, and HRF are biomarkers primarily linked to inflammation [114]. Steroids induce significant changes in inflammatory markers in the inner retina compared to anti-VEGF therapy, notably reducing HRF, DRIL extent, central retinal thickness (CRT), and cyst area [115]. Giant intraretinal cysts (>200 µm) and suspended scattering particles in motion are associated with macular ischemia and are considered indirect indicators of poor visual prognosis [13,118]. The top four OCT positive predictive factors for steroid responsiveness seem to be large cysts (>250 µm), DRIL, HRF and chronic SRF [101] (Figure 6). The chronicity of DME increases the likelihood of a suboptimal response to anti-VEGFs, which may explain the association between HRF in the outer retinal layers and SRF and disease recurrence [116]. Current international and national guidelines recommend initiating therapy with intravitreal steroids in patients presenting with these inflammatory biomarkers (Table 4) [96,129,130,131].

### 2.20. Discussion, Unmet Needs and Future Perspectives

The search for biomarkers in DME is critical, as they may influence the frequency of monitoring, guide the choice of an individualized therapeutic strategy, and ultimately help to avoid undertreatment, thereby affecting visual prognosis. Several limitations hamper the generation of robust evidence from serum, aqueous humour, or vitreous analyses, including small sample sizes, limited profiling of angiogenic and inflammatory mediators, and heterogeneous inclusion criteria. Furthermore, many studies compare patients with PDR to heterogeneous non-inflammatory or mixed-pathophysiology conditions—such as retinal detachment, vitreous hemorrhage, lens subluxation, and macular holes—assuming these entities to be appropriate control groups. Such assumptions introduce potential bias, compromise data interpretation, and ultimately reduce the overall validity of the findings [60,69,137,138,139].

There is broad consensus across studies that EZ disruption, DRIL, DCP loss, epiretinal membrane with retinal traction, and the presence of intraretinal cysts are all associated with a poorer visual outcome in DME. Among these, IRF is particularly refractory to anti-VEGF therapy and tends to respond less favourably than SRF, even with newer agents such as faricimab 6 mg and aflibercept 8 mg [140,141]. The above features are recognized as hallmarks of chronic DME, whereas SRF is often associated with more acute forms or acute recurrence. However, in chronic or recurrent DME, SRF may persist and become more challenging to manage [116,117]. In such cases, IRF may show greater responsiveness to steroids than to anti-VEGF agents [75]. The persistence of IRF in >40% of eyes under treatment with the newer anti-VEGFs highlights a significant unmet need in the management of DME [140]. Although steroids are effective in reducing retinal thickness, they have not consistently demonstrated superior BCVA gains compared to anti-VEGF agents. Nevertheless, earlier initiation on steroid therapy may improve outcomes, as eyes with a baseline BCVA ≥ 65 letters are 11 times more likely to reach a final BCVA ≥ 75 letters, suggesting that timely intervention may be critical [75].

Although the role of inflammation in DR and DME is well established, either by experimental or clinical studies, the association of biomarkers, namely OCT biomarkers, with the prognosis or with the choice to treat with steroid implants is not consensual [75,103,108,109,110,111,112,113,114,115]. Further robust, prospective evidence is needed to clarify the optimal timing and selection criteria for steroid use in DME, since most of the studies are small or retrospective and non-controlled [114,115,116,117,125,126,127]. HRF are recognized as inflammatory OCT biomarkers, but their role in predicting superior response to steroids over anti-VEGF agents in DME remains controversial. Some authors argue that no robust evidence currently supports HRF—or any other biomarker—as a reliable predictor of steroid responsiveness. Similarly, comparative evidence between steroids and anti-VEGF agents remains limited. For a biomarker to justify choosing steroids over anti-VEGFs, it must demonstrate an association with poor anatomical or functional outcomes under anti-VEGF treatment [142]. While secondary analyses of randomized trials have explored prognostic factors, none have confirmed predictive biomarkers for steroid benefit. However, younger age, less severe DR, and absence of surface wrinkling retinopathy on CFP have been associated with better visual prognosis, after adjusting for baseline BCVA. The presence of HRF correlated with increased CRT, SRF, IRF, cysts, and EZ disruption. However, no significant differences in BCVA, CRT, or HRF changes were observed between dexamethasone and ranibizumab treatment groups. HRF tend to be more frequent in poor responders and are considered markers of disease chronicity, rather than predictors of differential therapeutic response [143,144].

Likewise, the effectiveness of switching therapies in persistent DME remains a matter of ongoing debate. Evidence suggests that a suboptimal response at 12 weeks does not necessarily predict poor long-term outcomes. Indeed, many eyes with minimal early improvement (e.g., <5-letter gain) still achieved meaningful visual gains (≥10 letters) at 2 years without switching therapy [145]. In Protocol T, persistent centre-involved DME at 6 months was more common in eyes treated with bevacizumab than in those treated with aflibercept or ranibizumab. Nevertheless, mean BCVA gains at 2 years were similar regardless of early DME persistence or the anti-VEGF agent used [146]. Therefore, caution is warranted when considering a treatment switch after three or more injections, as continued improvement may still occur with ongoing therapy using the same agent. Importantly, ≥10-letter vision loss was rare among eyes with chronic persistent DME [146]. Furthermore, some expert consensus statements, including those from Italy [147] and the United Kingdom [148], have recommended a five-month loading phase of injections, consistent with the treatment regimens used in the VIVID and VISTA trials [149]. However, sub-analyses from DRCR.net Protocol I demonstrated that eyes with chronic persistent DME—defined as DME lasting ≥24 weeks despite monthly treatment with 0.5 mg ranibizumab—experienced significantly worse long-term visual outcomes than eyes with shorter-duration DME. Mean BCVA gain at 3 years was 7 letters in the chronic group compared with 13 letters in those with shorter-lasting edema [146]. These findings suggest that early functional response at 12 weeks is a reliable predictor of long-term outcome, as the visual gains observed at that stage were largely maintained over the 3-year follow-up [150]. Additionally, sub-analyses from DRCR.net Protocol I showed that eyes with a robust early anatomical response—defined as ≥20% reduction in CRT after three loading doses of ranibizumab—experienced significantly greater improvements in BCVA over 3 years to those with a limited response (<20%) [151].

A meta-analysis of 14 randomized clinical trials involving 827 eyes found no biomarkers capable of predicting differential responses between steroid and anti-VEGF therapies. Although CRT was significantly lower with steroids at 3 and 6 months and at final follow-up, no significant differences in BCVA were observed at 3, 6, or 12 months, or at the study endpoint [152]. Persistent IRF is likely associated with better responsiveness to steroids, as steroids are particularly effective at reducing macular fluid. Persistent IRF at 24 weeks or the presence of large cysts may therefore be strong indicators for earlier steroid initiation, potentially improving prognosis by shortening the duration of chronic persistent DME [101,140,141].

A recent systematic review and meta-analysis found that baseline HRF, hyperreflective choroidal foci, DRIL, and disruption of the EZ or ELM, and disrupted cone outer segment tips (COST) line were associated with worse BCVA, with a moderate level of certainty of evidence [112]. However, none of the evaluated biomarkers was associated with better BCVA with moderate certainty. Similarly, no biomarker demonstrated a high level of certainty of evidence for either improved or worsened BCVA. The authors highlighted substantial heterogeneity in current biomarker assessment methodologies [112]. Notably, hyperreflective choroidal foci are neglected in most studies, although they may correspond to glial cell migration, proliferation, and accumulation in the choroid, as previously described in the section on cellular biomarkers.

Even in the absence of robust evidence, the aforementioned data suggest that eyes with a poorer early response tend to achieve worse long-term visual outcomes. Therefore, earlier introduction of steroid therapy in cases of persistent DME may be warranted, to determine whether more rapid resolution of macular edema leads to improved final BCVA [153,154,155,156].

This review has several important limitations that should be acknowledged. First and foremost, it is a narrative rather than a systematic review, and the authors did not formally classify the level of evidence of the included studies. Explicit inclusion and exclusion criteria were not reported, nor were the numbers of records identified, screened, excluded, and included (a PRISMA-style flowchart was not provided). Consequently, the risk of bias cannot be excluded. In addition, studies employing different therapeutic agents and treatment regimens were included, as were studies using heterogeneous approaches to grading OCT biomarkers. Another limitation is the lack of paraclinical investigations such as adaptive optics, microperimetry and ERG as predictive factors or outcome parameters.

The strengths of this review lie in its comprehensive overview of the published literature and its critical appraisal of the available evidence regarding the value of OCT biomarkers in DME. The nationwide, multicentre participation of the study further strengthens its relevance. Importantly, the critical perspective presented in this review is consistent with the conclusions of recent systematic reviews and meta-analyses.

## 3. Conclusions and Future Perspectives

Biological markers and retinal imaging biomarkers underscore the critical role of inflammation in the pathophysiology of DR and DME. Prognostic and predictive biomarkers integrating both morphological and functional parameters could enable personalized treatment strategies, potentially improving visual outcomes, facilitating earlier detection of disease progression, and supporting more effective interventions to delay or prevent vision loss. However, the identification of reliable biomarkers to guide treatment selection in DME remains controversial. Most biomarkers associated with poor visual outcomes under anti-VEGF therapy—such as HRF, DRIL, or EZ disruption—primarily reflect disease chronicity rather than treatment-specific responsiveness. To date, no adequately powered prospective studies have demonstrated that these biomarkers predict superior outcomes following intravitreal anti-VEGF or steroid therapy. Moreover, the clinical integration of intraocular biomarkers obtained from aqueous or vitreous samples is constrained by the lack of standardized sampling protocols, unclear interpretative thresholds, and insufficient evidence regarding cost-effectiveness and impact on clinical outcomes. Consequently, biomarker-driven treatment algorithms in DME remain a promising yet unrealized concept that requires further validation in rigorously designed clinical trials. Despite the absence of robust biomarker-guided evidence, substantial clinical data indicate that corticosteroids are the most effective agents for achieving rapid macular drying. Therefore, their earlier introduction may be justified in cases of persistent IRF at 24 weeks or in the presence of large cystoid spaces, as shorter duration of macular edema is likely associated with better final BCVA. A further critical limitation is the lack of unequivocal in vivo evidence directly correlating HRF observed on OCT with microglial cell accumulation demonstrated in ex vivo or in vitro models. Artificial intelligence (AI) may play a pivotal role in bridging this gap by facilitating the identification of correlations between imaging biomarkers and treatment response in DME. In particular, AI-based models could help link in vivo OCT features—such as HRF—with microglial activation observed in animal models or post-mortem human tissue, thereby helping to exclude alternative explanations for the origin of hyperreflective signals on OCT.

Future research should prioritize large, methodologically robust randomized clinical trials aimed at clearly defining both positive and negative prognostic factors in DR and DME. Achieving this goal will require the establishment of validated, standardized, and universally accepted definitions for each OCT and OCTA biomarker. Currently, several biomarkers—such as DRIL—remain difficult to classify consistently across studies, while EZ integrity outcomes exhibit substantial heterogeneity. Clear outcome definitions, standardized biomarker grading, and determination of the relative contribution of each biomarker to visual function are essential. Furthermore, the association between specific biomarkers and the need for treatment escalation or switching to steroid implants must be evaluated in an unbiased manner. Only through such an approach can patient and clinician expectations be effectively guided, ultimately informing monitoring strategies, treatment intervals, and therapeutic selection.

## Figures and Tables

**Table 1:** Vitreous biomarkers.

Biomarker Class	Name of Biomarker	Result	Reference
Biomarker of oxidative stress	Malondialdehyde	Patients with PDR demonstrated significant levels of vitreous malondialdehyde.	Brzović-Šarić V et al., 2015 [28]
Angiogenic factors	VEGF	Aqueous and vitreous VEGF were significantly higher in patients with DME compared to healthy controls.	Minaker et al., 2022 [29]
	PIGF	PIGF was detected in the vitreous samples of DR patients.	Khaliq et al., 1998 [30]
	Erythropoietin	DME patients showed higher vitreous concentrations of erythropoietin compared to control patients.	Garcí-Arumí J et al., 2009 [31]
	Angiopoietin	DME patients demonstrated a slight increase in the intravitreal concentration of angiopoietin versus the control group.	Joussen AM et al., 2002 [32]
	MMP-9	PDR patients exhibited significantly higher levels of MMP-9 in the vitreous compared to non-diabetic control patients.	Kowluru et al., 2012 [33]
Pro-inflammatory cytokines	Interleukin	PDR patients revealed significantly higher levels of IL-6 and IL-8 compared to control patients.	Takahashi et al., 2016 [34]
	TNF-α	PDR patients showed increased TNF-α levels compared to control patients. TNF-α increased with the severity of the disease.	Costagliola et al., 2013 [35]
	Receptor tyrosine kinase	PDR patients demonstrated higher levels of the receptor tyrosine kinase.	Abu El-Asrar et al., 2013 [36]
	IL-10	PDR patients exhibited increased IL-10 concentration compared to the control group.	Lopez-Contreras et al., 2020 [37]
	PDGF-AA, TGF-α, VEGF, IL-6, IL-8, and TNF-β	Patients with proliferative PVR showed a significant increase in PDGF-AA, TGF-α, VEGF, IL-6, IL-8, and TNF-β levels.	Ni et al., 2020 [38]
	TGF-β	PDR patients exhibited higher levels of TGF-β.	McAuley et al., 2013 [39]
	IL-1β	PDR patients demonstrated higher levels of IL-1β and IL-10 compared to the control group.	Mao C & Yan H, 2014 [40]

DR = diabetic retinopathy; IL-1β = interleukin 1 beta; IL-6 = interleukin 6; IL-8 = interleukin 8; IL-10 = interleukin 10; MMP-9 = matrix metalloproteinase 9; PDR = proliferative diabetic retinopathy; PDGF-AA = platelet-derived growth factor AA; PIGF = placental growth factor; PVR = proliferative vitreoretinopathy; TGF-α = transforming growth factor alpha; TGF-β = transforming growth factor beta; TNF-α = tumour necrosis factor alpha; TNF-β = tumour necrosis factor beta; VEGF = vascular endothelial growth factor.

**Table 2:** Tear fluid biomarkers in patients with DME.

Tear Fluid Biomarker	Study Inference	Reference
IP-10, MCP-1, and angiogenic cytokines	Association of chronic inflammatory reaction and angiogenesis in the ocular surface of diabetic patients.	Li et al., 2010 [79]
Lipocalin 1, lactotransferrin, lacritin, lysozyme C, lipophilic A, and immunoglobulin lambda chain	Patients with DR showed relatively higher levels of the stated biomarkers.	Csősz et al., 2012 [80]
AGE product proteins such as lysozyme, lactoferrin, and lactocalin	AGE-modified proteins may be utilized as biomarkers for the diagnosis of DR.	Nishikawa et al., 2000 [23] Du et al., 2000 [24]
TNF-α	Higher levels of TNF-α were strongly correlated with DR severity.	Costagliola et al., 2013 [35]

AGE = advanced glycation end-products; DR = diabetic retinopathy; IP-10 = interferon-induced protein 10; MCP-1 = monocyte chemoattractant protein 1; TNF-α = tumour necrosis factor alpha.

**Table 3:** Application of OCT biomarkers in patients with DME.

Objective of the Study	Imaging Modality	Inference	Reference
To assess the occurrence of HRF in diabetic patients without clinically observable retinopathy or presence of mild to moderate retinopathy without DME	SD-OCT	Presence of HRF in eyes of diabetic patients even when clinical retinopathy was undetectable	Vujosevic et al., 2013 [104]
Comparison of the morphologic and functional features of DME with and without SND	CFP, FA, SD-OCT, BCVA, and microperimetry	The observed data could help characterize DME in eyes with SND	Vujosevic et al., 2017 [100]
Comparison of the therapeutic effects of intravitreal ranibizumab and DEX utilizing SS-OCT retinal biomarkers in DME patients	BCVA and SS-OCT	Ranibizumab and DEX were effective in the treatment of DME, as evidenced by functional enhancement and morphological biomarker changes	Ceravolo et al., 2020 [105]
Comparison of concentration of aqueous angiogenic and inflammatory cytokine in DME patients	OCT	The levels of aqueous cytokines varied according to the morphological characteristics of DME. There is a requirement of both anti-angiogenic and anti-inflammatory agents for DME	Bandyopadhyay et al., 2018 [106]

BCVA = best-corrected visual acuity; DME = diabetic macular edema; DEX = dexamethasone; FA = fluorescein angiography; CFP = colour fundus photography; HRF = hyperreflective foci; OCT = optical coherence tomography; SD-OCT = spectral domain optical coherence tomography; SND = subfoveal neuroretinal detachment; SS-OCT = swept source optical coherence tomography.

**Table 4:** Role of various inflammatory biomarkers in response to DME treatment.

Objective of the Study	Result	Inference	Reference
Assessment of the correlation between AH levels of cytokines/growth factors and the response of treatment regimen (intravitreal ranibizumab) for DME	Patients who responded well to the treatment showed significantly higher levels of baseline aqueous concentrations of VEGF, PlGF, sVEGFR1, MCP-1, ICAM-1, IL-6 and IP-10	The aqueous concentration of VEGFR1 family members and various inflammatory cytokines tend to be correlated with responses to ranibizumab therapy for DME	Shimura et al., 2017 [132]
Evaluation of specific changes in retinal imaging biomarkers as suitable indicators of retinal inflammatory condition	DEX-treated patients showed a slightly higher decline in CMT than ranibizumab-treated patients. Higher baseline HRF numbers and the presence of IFAF were associated with greater improvements in retinal sensitivity in the DEX group. Moreover, patients with SND at baseline had a significant decrease in CMT relative to those without SND	An increased level of HRF, a larger IFAF region, and persistent SND could point to a common inflammatory condition in DME that responds well to targeted treatment	Vujosevic et al., 2017 [133]
Assessment in the changes in short-term HEs following intravitreal triamcinolone, DEX implant, or bevacizumab injections in DME patients	Patients receiving triamcinolone or DEX implant demonstrated a significant reduction in HEs after one and two months, respectively	Intravitreal steroids may be a suitable treatment regimen in DME patients with HEs in the fovea	Shin et al., 2017 [134]
Evaluation of correlation between systemic pro-inflammatory factors and macular structural response to intravitreal bevacizumab in DME patients	Serum MCP1 and serum VEGF showed significant relationship with a 25% decline in CFT at a time span of 6 months. Both hsCRP and ICAM1 had a significant relationship with a 10% reduction in third month CFT	Higher levels of hsCRP and ICAM1 revealed to be relevant biomarkers for anatomic response to anti-VEGF therapy	Brito et al., 2018 [135]
Comparison of the efficacy and safety of intravitreal DEX implant versus aflibercept in patients with treatment-naïve DME with inflammatory biomarkers	DEX implant and aflibercept groups had a significant improvement in visual acuity at the end of the follow-up period (12 months)	Both DEX implant and aflibercept were effective and safe in treatment-naïve DME patients with inflammation	Ozsaygili et al., 2020 [136]
Review Study The role of Intravitreal corticosteroids in the treatment of DME: predictive OCT biomarkers	This work summarizes predictive biomarkers to guide treatment choice in DME	OCT parameters such as large amount of retinal and choroidal HRF, disruption of the outer retinal layers, and other markers of chronicity including intraretinal cysts extending into the outer retina, a reduced choroidal vascular index or the presence of SRF in chronic DME, may predict a more favourable response to steroids compared to anti-VEGF	Munk et al., 2022 [101]
Quantitative assessment of functional and structural features in nonvitrectomized and vitrectomized DME patients after being treated with an FAc implant	Patients who needed additional therapy after FAc had a higher prevalence of SRF and CFT at baseline	The presence of SRF and increased CFT in non-responders to anti-VEGF, together with a limited short-term response to CCT, have been associated with a lower likelihood of sustained response to long-term CCT and may be important indicators of the requirement for additional therapeutic intervention	Pessoa et al., 2021 [117]
Longitudinal analysis of the effect of OCT biomarkers on macular thickness in patients with persistent macular edema secondary to diabetes mellitus and retinal vein occlusion who received intravitreal DEX implant	In the presence of HRF and SMD, recurrence of macular edema was significant in the 3rd month	DEX implant therapy resulted in a satisfactory reduction in CFT in patients with DME and RVO. The presence of HRF and SMD was a negative predictor of recurrence in CFT with DEX implant and resulted in satisfactory improvement in SMD	Horozoglu et al., 2023 [116]

AH = aqueous humour; CFT = central foveal thickness; CMT = central macular thickness; CRP = C-reactive protein; DEX = dexamethasone; DME = diabetic macular edema; FAc = fluocinolone acetonide; HE = hard exudates; HRF = hyperreflective foci; hsCRP = high-sensitivity C-reactive protein; ICAM-1 = intercellular adhesion molecule-1; IFAF = fundus autofluorescence images; IL-6 = interleukin 6; IP-10 = inducible protein-10; MCP-1 = monocyte chemoattractant protein-1; OCT = optical coherence tomography; PlGF = placental growth factor; RVO = retinal vein occlusion; SMD = serous macular detachment; SND = subfoveal neuroretinal detachment; SRF = subretinal fluid; sVEGFR1 = soluble VEGF receptor-1; VEGF = vascular endothelial growth factor.

## Data Availability

No new data were created or analyzed in this study.

## Abbreviations

AGEs	Advanced glycation end-products
AH	Aqueous humour
Ang	Angiopoietin
AQP	Aquaporin
BCVA	Best corrected visual acuity
BRB	Blood-retinal barrier
CFT	Central foveal thickness
CFP	Colour fundus photography
CME	Cystoid macular edema
CRP	C-reactive protein
CRT	Central retinal thickness
DCP	Deep capillary plexus
DEX	Dexamethasone
DME	Diabetic macular edema
DR	Diabetic retinopathy
DRIL	Disorganization of the retinal inner layers
ERM	Epiretinal membrane
ETDRS	Early Treatment Diabetic Retinopathy Study
EZ	Ellipsoid zone
FA	Fluorescein angiography
FAF	Fundus autofluorescence
FAZ	Foveal avascular zone
FAc	Fluocinolone acetonide
GAPDH	Glyceraldehyde-3-phosphate dehydrogenase
GRO	Growth regulated oncogene
HbA1c	Glycated hemoglobin
HE	Hard exudates
HGF	Hepatocyte growth factor
HRF	Hyperreflective foci
HsCRP	High-sensitivity C-reactive protein
ICAM-1	Intercellular adhesion molecule-1
iFAF	Increased FAF
IL	Interleukin
INL	Inner nuclear layer
IP-10	Interferon-gamma-induced protein 10
IRF	Intraretinal fluid
MA	Microaneurysm
MCP-1	Monocyte chemoattractant protein-1
MIG	Monokine induced by gamma interferon
MMP-9	Matrix metalloproteinase 9
NF-kB	Nuclear factor-kappa B
NPDR	Non-proliferative diabetic retinopathy
NT	Neurotrophins
OCT	Optical coherence tomography
OCTA	Optical coherence tomography angiography
OPL	Outer plexiform layer
PDGF	Platelet-derived growth factor
PDR	Proliferative diabetic retinopathy
PEDF	Pigment epithelium-derived factor
PlGF	Placental growth factor
PKC	Protein kinase C
RANTES	Regulated on Activation, Normal T Cell Expressed and Secreted
RPE	Retinal pigment epithelium
SD-OCT	Spectral-domain optical coherence tomography
SRF	Subretinal fluid
T1DM	Type 1 diabetes mellitus
T2DM	Type 2 diabetes mellitus
TGF	Transforming growth factor
TNF	Tumour necrosis factor
VCAM-1	Vascular cell adhesion molecule-1
VD	Vascular density
VEGF	Vascular endothelial growth factor
WF	Widefield
